# Deep Learning-Based Haptic Guidance for Surgical Skills Transfer

**DOI:** 10.3389/frobt.2020.586707

**Published:** 2021-01-20

**Authors:** Pedram Fekri, Javad Dargahi, Mehrdad Zadeh

**Affiliations:** ^1^Mehchanical, Industrial, and Aerospace Engineering Department, Concordia University, Montreal, QC, Canada; ^2^Electrical and Computer Engineering Department, Kettering University, Flint, MI, United States

**Keywords:** deep learning, recurrent neural network, LSTM, haptic, force feedback, bone drilling, surgical skill transfer, COVID-19

## Abstract

Having a trusted and useful system that helps to diminish the risk of medical errors and facilitate the improvement of quality in the medical education is indispensable. Thousands of surgical errors are occurred annually with high adverse event rate, despite inordinate number of devised patients safety initiatives. Inadvertently or otherwise, surgeons play a critical role in the aforementioned errors. Training surgeons is one of the most crucial and delicate parts of medical education and needs more attention due to its practical intrinsic. In contrast to engineering, dealing with mortal alive creatures provides a minuscule chance of trial and error for trainees. Training in operative rooms, on the other hand, is extremely expensive in terms of not only equipment but also hiring professional trainers. In addition, the COVID-19 pandemic has caused to establish initiatives such as social distancing in order to mitigate the rate of outbreak. This leads surgeons to postpone some non-urgent surgeries or operate with restrictions in terms of safety. Subsequently, educational systems are affected by the limitations due to the pandemic. Skill transfer systems in cooperation with a virtual training environment is thought as a solution to address aforesaid issues. This enables not only novice surgeons to enrich their proficiency but also helps expert surgeons to be supervised during the operation. This paper focuses on devising a solution based on deep leaning algorithms to model the behavior of experts during the operation. In other words, the proposed solution is a skill transfer method that learns professional demonstrations using different effective factors from the body of experts. The trained model then provides a real-time haptic guidance signal for either instructing trainees or supervising expert surgeons. A simulation is utilized to emulate an operating room for femur drilling surgery, which is a common invasive treatment for osteoporosis. This helps us with both collecting the essential data and assessing the obtained models. Experimental results show that the proposed method is capable of emitting guidance force haptic signal with an acceptable error rate.

## 1. Introduction

Lack of having an appropriate medical training system may cause errors with adverse effects on patients. The practical intrinsic of medical education systems has led expert surgeons to transfer their skill to trainees via trial and error methods in the actual operating rooms. Evidently, novice surgeons have a minuscule chance of the repetition for improving their proficiency during the operation. Complications through surgical procedures have considerably raised, which signifies young surgeons require to be more proficient. On the one hand, achieving hands-on skills in an actual operation room is a tedious and time-consuming process. On the other hand, training in operative rooms is extremely expensive so that estimations reveal a significant increase in operative time for training translated into about $53 million dollars per year (de Montbrun and MacRae, 2012). However, the National Health Service (NHS) has recommended a restriction in working hours for trainees (from 30,000 working hours to around 7,000 h) to increase the effective time of expert surgeons as well as improve the outcome of surgeries (Tan and Sarker, 2011). The aforementioned limitations accentuate the importance of having a proper educational system for surgeons other than the traditional methods.

Apart from the above challenging issues, the circumstances such as the recent global shutdown due to the COVID-19 pandemic cause a closure in the education system as well. In this case, trainees find it tough to attend the in-person sessions and operation rooms for educational purposes. On the one hand, most of operations are deferred in order to reduce the burden on the shoulders of hospitals' staff. On the other hand, enacting preventive rules and initiatives such as social distancing brings about encountering with the limitation in using surgical labs (Al-Jabir et al., 2020). To this end, in order to maintain surgical skills, the demands for using simulations and artificial intelligence methods have been soared. For instance, neurosurgical residents in New Orleans have been encouraged to utilize the aforementioned technologies so as to practice complex surgical task during the COVID-19 pandemic. The same concern has emerged from the community of orthopedics surgeons so that they have tended to use surgical simulation for their residents. Hence, it is necessary to equip the medical education system in such a way that the remote working become conceivable (Bernardi et al., 2020).

Although the above issues can be generalized to a wide range of surgical operations, for the sake of simplicity, this work exclusively concentrates on the hip fracture treatment. One of the most common health issues is hip fracture that is seen in elderly adults with a high mortality rate of 20 and 35% within 1 year (Goldacre et al., 2002; Thorngren, 2008). The cause, on the other hand, chiefly stems from osteoporosis so that 2–8% of males and 9–38% of females are diagnosed with this disease, which accounts for overall 30 million women and 8 million men around the United States and EU (Schapira and Schapira, 1992; Svedbom et al., 2013; Wade et al., 2014; Willson et al., 2015). Therefore, the hip fracture issue needs a precise and reliable treatment regardless of patients' gender.

Closed reduction percutaneous pinning (CRPP) is a typical treatment for supporting hip fractures, in which surgeons perform based on hands-on experiences in the operating room. This type of treatment is invasive and needs professional surgeons to do the task, thereby diminishing the presumable complications. Since the probability of making inadvertent mistakes is high, expert surgeons are mostly reluctant to operate and instruct novices simultaneously. With this in mind, having an auxiliary system is necessary so as to overcome the challenges and reduce the risk of medical errors as well as simplify the training in medical education systems. This leads to not only enhance the novice hands-on skills but also supervise expert surgeons during the surgery.

Skill transfer system is considered as a solution with the aim of addressing the aforesaid complications. As a matter of fact, the system models the performance of experts for a certain surgical procedure that culminates to a trained medical robot for fulfilling multiple goals such as more reliable assisting. An example of medical assistant robot is the human–robot interaction system, which has been introduced as a tool for improving human performance (Ramón Medina et al., 2012; Medina et al., 2015; Gil et al., 2019; Pezent et al., 2019). In general, a haptic device along with a simulation environment is deemed as an experimental setup for the human–robot interaction system in the surgical application. On the one hand, users manipulate the haptic device to complete a surgical task via a collaborative environment. On the other hand, haptic guidance signal is generated to correct or elevate the users' performance (Morris et al., 2006; Rozo et al., 2013). This setup can be utilized for the purpose of skill transfer in order to help experts to convey their knowledge in a safer environment.

In addition to the mechanical setup, it needs to elicit robust models from expert surgeons' demonstrations based on their dynamic and non-linear behaviors during the surgery. These behaviors are categorized into kinesthetic and kinematic demonstrations. Learning kinematic demonstrations is chiefly regarded as the process of extracting positional body movements of the expert during the operation (Abbott et al., 2007; Chipalkatty et al., 2011; Zahedi et al., 2017). Kinesthetic information can be obtained by a physical interaction between robots and users so that a surgeon directly works with the robot to perform a specified task (Rozo et al., 2013, 2016; Kronander and Billard, 2014).

All in all, the system comprises a perceptual part in conjunction with a robotic actuator in order to provide the realistic sense of surgery. In other words, this solution is an assistant human-in-the-loop system that consumes the data corresponding to either kinesthetic or kinematic demonstrations. The data are captured while the expert is manipulating the haptic end-effector to accomplish a specific task through a virtual environment. Then, the compiled dataset is used to train models for extracting experts' behaviors. Finally, the haptic guidance signal, which is emitted from the trained model, is employed as a reference signal for both trainees and experts. The trainees can correct their movements based on the provided signal through the virtual environment. The haptic guidance signal can be utilized as a supervisor for experts during a real operation as well.

Since the data related to both kinematic and kinesthetic demonstrations is time varying and also learning the performance of experts depends on modeling the dynamic behaviors over time, it is crucial to investigate the data with respect to the temporal characteristic. Statistical algorithms such as HMM and CRF have been used to extract the aforementioned features (Reiley and Hager, 2009; Ramón Medina et al., 2012; Tao et al., 2013; Zahedi et al., 2017, 2019). In addition, control algorithms have been applied to the human-in-the-loop system for guiding human via robots based on the model obtained from dynamic data (Chipalkatty et al., 2011; Safavi and Zadeh, 2015, 2017; Safavi et al., 2015). However, previous works have an impressive progress in modeling the dynamic data in the skill transfer system, advent of machine learning and deep learning algorithms is thought as a gigantic step toward developing more trustworthy predictive systems. For instance, a method has been proposed based on deep learning to predict the haptic feedback in percutaneous heart biopsy for decreasing delay in remote operations (Khatami et al., 2017). The temporal facet of data can help to enrich the throughput of the system whether in the data compilation or in the inference phase.

In this work, we seek to advance a solution for training novice residents in the orthopedic surgical drilling procedure by developing a skill transfer system using a deep learning method. The proposed system aims at contributing to the educational system of orthopedic residents during the COVID-19 pandemic. To this end, first, a simulation environment is used to visualize all components of a real operation room. The simulation creates a 3D visualization of the patient-specific bones from CT scan data. Moreover, it calculates an approximation of both bones' density and stiffness as physical properties of the tissue through the layers. As discussed in the next section, having these features helps to estimate the applied force feedback, when drill touches the bone. Using provided features, the system captures the essential data while expert surgeons perform a specific drilling task via the haptic device and simulation. Second, the solution aims to extract the model of expert surgeons' behaviors as a reference signal using the captured data. For this reason, a recurrent neural network with an LSTM architecture has been designed and implemented in order to be trained on force demonstrations as well as kinematic features and other effective data, which stems from either drill physics or bone tissues.

The main contributions of the proposed solution are summarized as follows: it investigates the influence of a deep recurrent neural network with an LSTM architecture on enhancing the quality of transfer skills in orthopedic surgical drilling. In contrast to the proposed solution in Khatami et al. (2017), our method incorporates multiple effective features, instead of utilizing force data solitary. In fact, in Khatami et al. (2017) the solution anticipates forces of X and Y directions using only the previously applied forces in the same directions and it does not engage other effective parameters in the estimation of force signals over time. Our proposed method, on the other hands, extracts the temporal behavior of force feedback data by fusing the data stemmed from multiple sources such as “bone's layer type,” “penetration depth,” “drill's temperature,” and “drill's position.” As a sensor fusion model, it will learn how to regulate the forces based on a fusion of data that impacts on the maneuver of the surgeon. Helping trainees to sense the guidance signal that stems from expert surgeons' hands-on skills along with other effective factors during surgery is another advantage of the proposed method. Also, since the force sensor is not utilized in this study, simulation aids to estimate forces as well as the other data.

The rest of the paper is organized as follows: the general explanation of proposed method along with experimental setup and data gathering are presented in section 2. Section 3 explains data preparation, computer experiments, result, and discussion. The paper concludes in the final section.

## 2. Deep Haptic Guidance Generator

**Figure 1:**
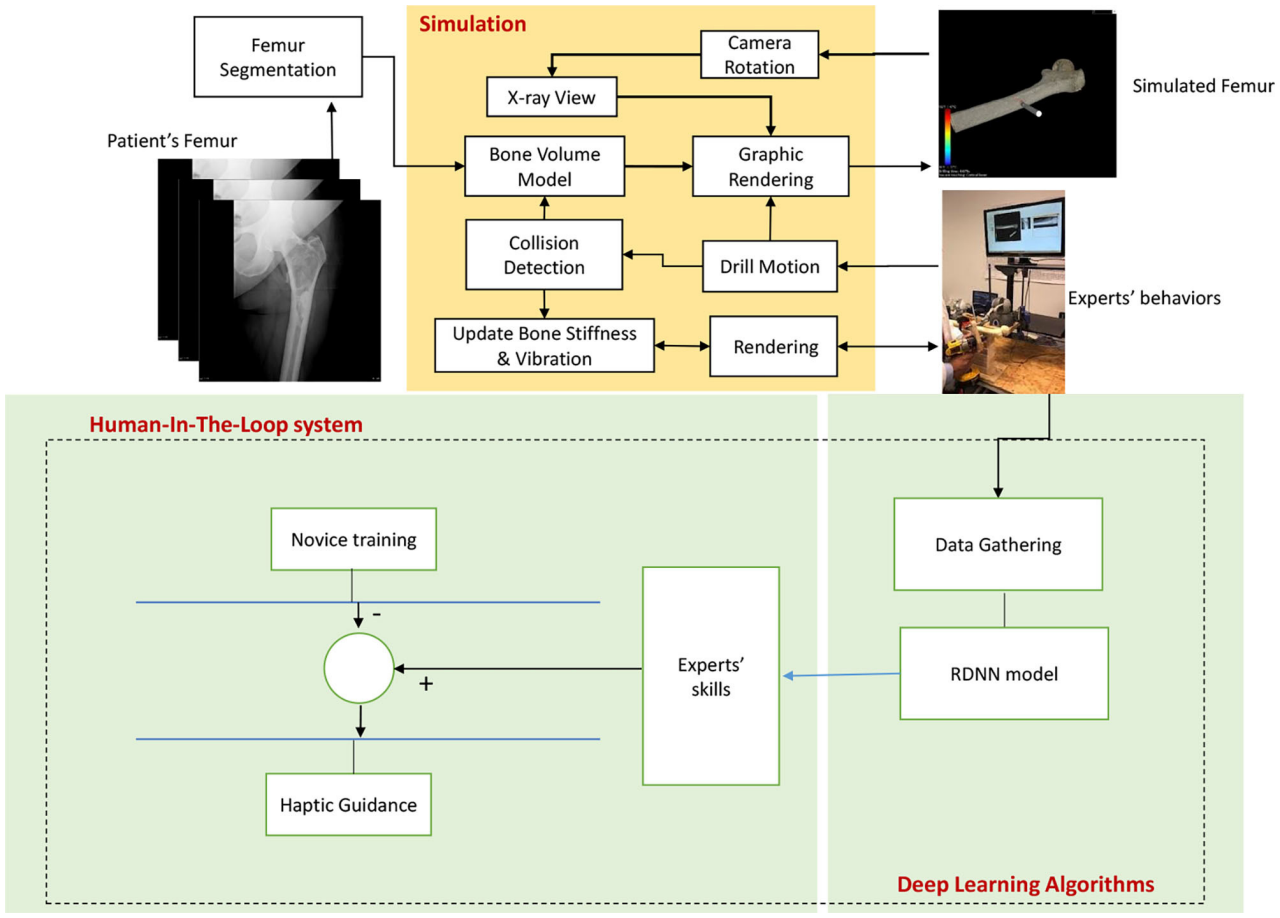
The diagram depicts the structure of the Deep Haptic Guidance (DHG). The CT data corresponding to a patient enters for modeling, rendering, and determining the stiffness of the bone. The data are captured while an expert is completing the task. The obtained data are converted to a compatible dataset for training deep recurrent neural network. The trained model provides haptic force feedback based on the professional demonstrations. The guidance signal is generated by the difference between the DHG's prediction and force feedback of novice user. In fact, expert's skill implies DHG's prediction.

The proposed system provides a solution based on a deep learning algorithm for conveying experts' hands-on proficiency to novice surgeons, exclusively in orthopedic surgical drilling with the purpose of treating hip fractures (Figure 1). This is achieved by utilizing a simulation environment, which creates a virtual shape of the intended patient's bone using CT scan images. This shape preserves the most principal features of the bone such as stiffness of every layer. At the same time, a haptic device is used by a user to drill a specified path through the bone. To provide a realistic sense, physical features such as temperature and rotation speed are considered, while the drill touches bone. This setup has two main advantages for the proposed method: First, it helps to capture the requisite data from expert surgeons while accomplishing a predefined drilling task. Second, it aids to exploit the trained model virtually.

Subsequently, a deep neural network is fed by the attained data to generate models on the behaviors of experts in a predefined task. As with the behavior modeling, learning gestures occurs over time. With this in mind, a recurrent neural network with an LSTM architecture is employed to extract a dynamic model. The aforementioned network is trained by the data of multiple sources (explained in detail in section 2.2) and then the force feedback in three axes is expected in its output. In other words, the proposed method with a modified LSTM architecture attempts to identify the dynamic relationship between inputs and their corresponding outputs in a supervised manner, while they are not equal in terms of dimension size. Intuitively, the significant objective is to investigate the impact of physical properties emerged from the bone's tissues or drill on the performance of the skill transfer system. As a sensor fusion system, however, this method outputs force feedback in three axes, it combines multiple data such as drill temperature, the type of bone's layer, penetration depth, and drill position rather than only force feedback as the network's input.

It is worth noting that, because we do not utilize force sensor, the corresponding data are estimated using the physical properties of the patients' bone obtained from CT images. For simplicity, we call the proposed method “DHG” (Deep Haptic Guidance). Figure 1 illustrates the structure of the DHG. Experimental setup, data gathering, and DHG are explained in the following sections.

### 2.1. Simulation and Experimental Setup for Bone Drilling Surgery

**Figure 2:**
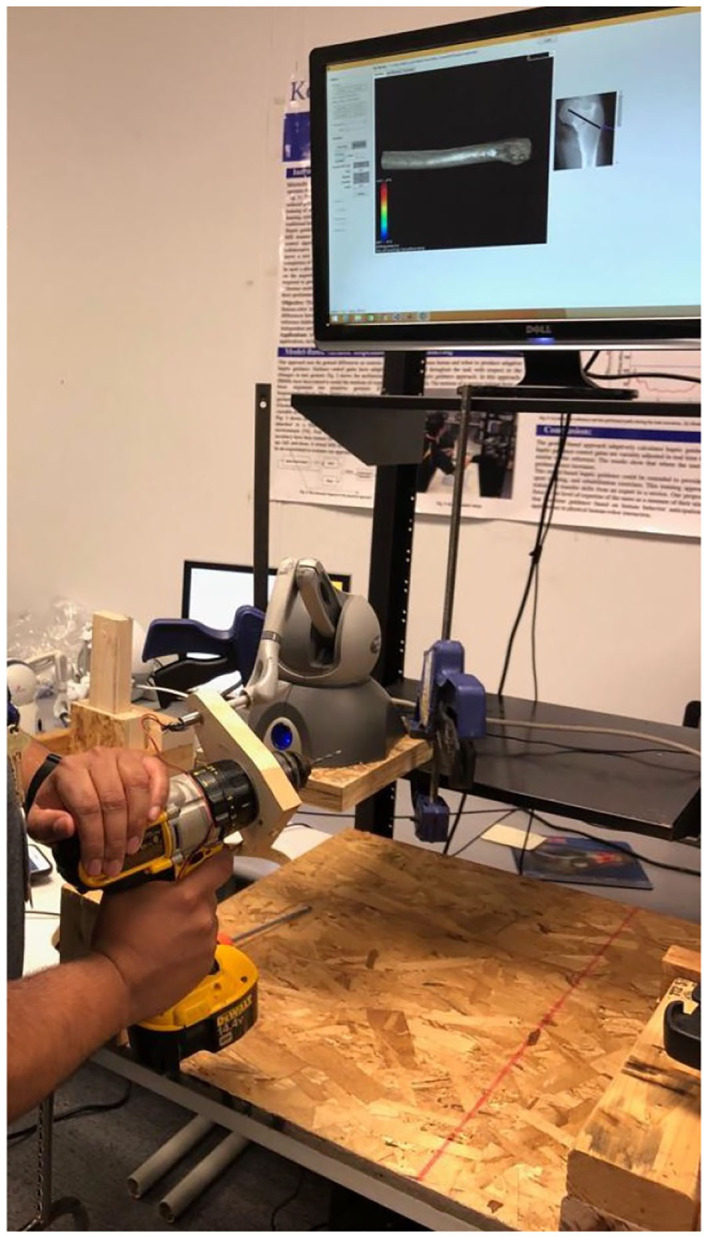
Experimental setup: A volunteer is completing a required surgical drilling task while using a drill attached to the haptic device. It encompasses a haptic device, a keyboard, and a computer mouse interacting with a simulation.

An experimental setup is used so as to capture the required data. It encompasses a haptic device (Phantom Omni, Geomagic Touch, USA) and a virtual environment (VE). The haptic device was set up on a movable and height adjustable table. The height of the table can be adjusted with respect to the convenience of the participants. The surgeons either experts or novices interact with the simulation system via the haptic device, a keyboard, and a computer mouse. A virtual drill can be manipulated to touch/drill the femur bone's shape through the stylus of the haptic device. The haptic device records motions of the end-effector, while it has been attached to a drill (Figure 2). It has 6 Degree of Freedom (DOF) positional sensing and capable of providing the user with the force feedback in 3 DOF translational motions (the space of force feedback data). The sampling frequency for compiling data is 10 Hz. The estimation on stiffness of the bone's layer is updated every 20 ms. The tasks were completed by moving the end-effector in order to drill and penetrate through the bone along the pre-defined path.

**Figure 3:**
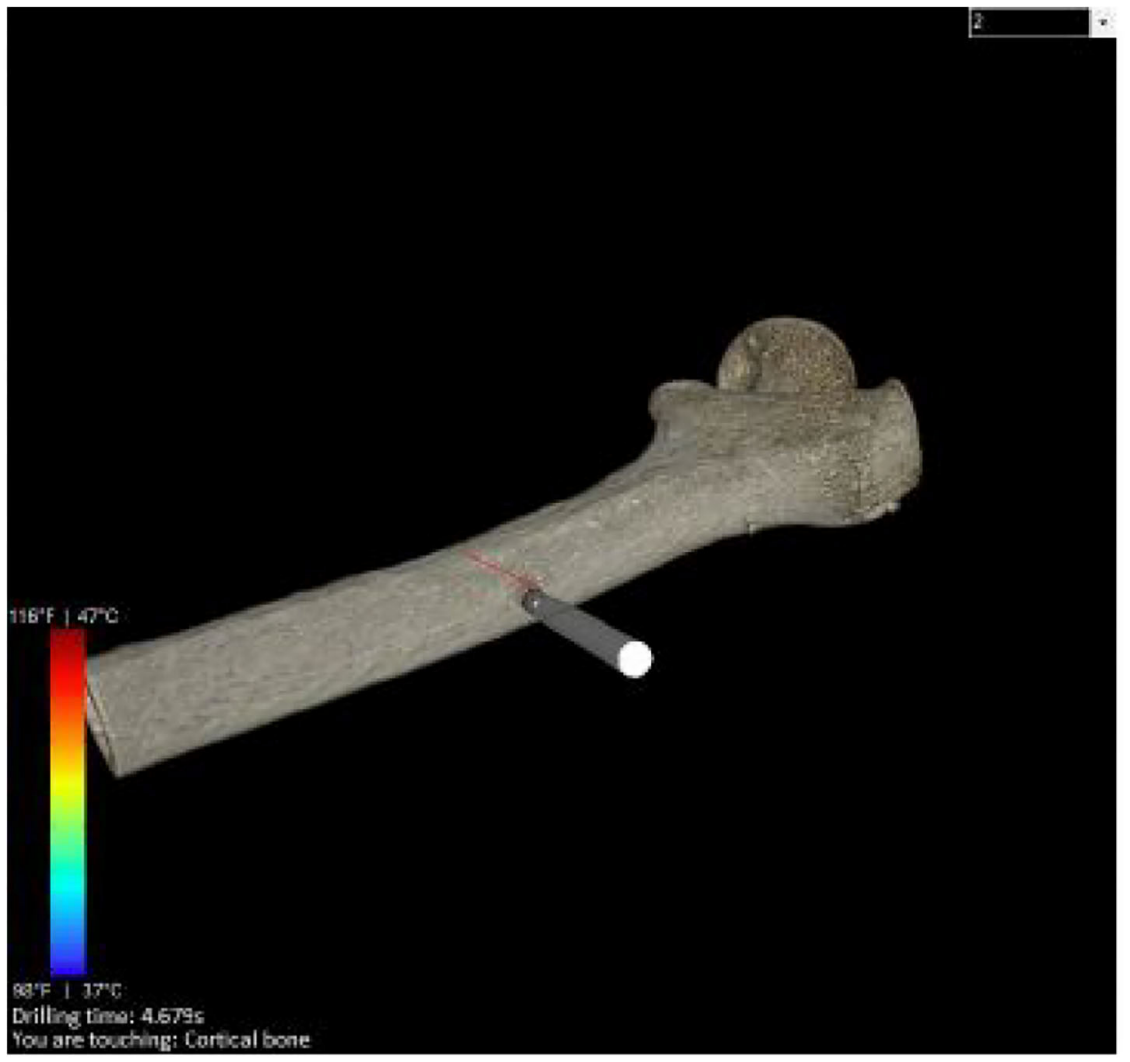
The image shows a rendered bone obtained from the CT data. The user is able to touch or drill the shape, visually by moving the object sticked the bone.

**Figure 4:**
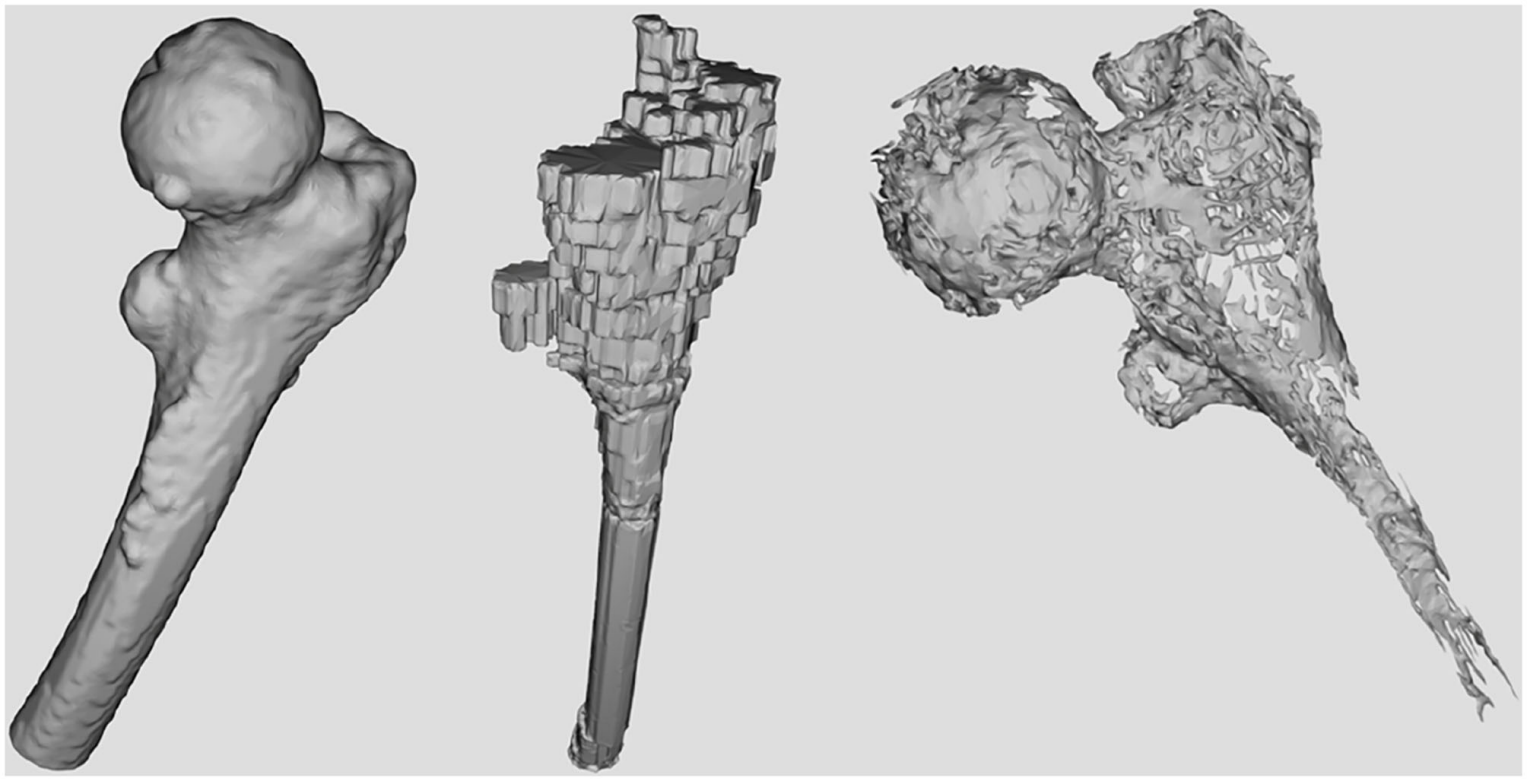
The surfaces corresponding to the bone's layers extracted from CT data. From left to right: Cortical bone surface, bone marrow, and necrosis.

In this setup, the simulation plays a crucial role in capturing data. Generally, simulated and virtual reality (VR) environments can be considered as an alternative of real operation rooms (Seymour, 2008; Kho et al., 2015; Van Duren et al., 2018). This enables trainers to define particular surgical task and drilling path for each patient separately. Here, we simulated attributes of the bone in order to gather data from experts during a surgery. For more precise explanations, four main parts of a required simulation, exclusively for bone drilling, are described as follows:

First, as shown in Figure 1 it needs to simulate and render a patient's femur bone by considering physical properties. This is obtained by using the patient's specific CT images as the input of the simulation (Figure 3). Since the goal is to model the physical characteristics of the femur bone related to a patient with femoral head necrosis, CT is more useful than MRI images (Teo et al., 2006). Furthermore, as shown in Figure 4, a segmentation method ascertains different layers such as cortical bone, cancellous bone, and bone marrow in order to simulate their thickness, stiffness, and physical traits while interacting with a drill. The intensity values of the CT images are utilized to segment the bone's layers along with their mechanical properties. Namely, the bright part of a CT image is cortical bone, whereas the dark parts are cancellous bone.

Second, to make more actual sense of working with the simulator, a method is employed to change the virtual shape of the bone, whenever the drill touches or penetrates the tissue. In other words, the method models stiffness of tissues based on the bone's depth and then assigns a specific value to every voxel in each layer of the bone. Finding the relationship between intensity of voxels and density of the bone in the images helps the volume rendering operation to take an appropriate action. In fact, the rendering operation decides to keep or remove a voxel with respect to its density value and the drill status as well (Morris et al., 2004; Liu and Laycock, 2009; Sofronia et al., 2012; Bogoni and Pinho, 2013).

Third, the mechanical traits of the obtained layers are involved to simulate haptic force feedback. As a result, cortical bone is much more stiffer that cancellous bone (Compere, 1980). On the other hand, the cancellous bone of the femur bone has a wide range of density that affects drilling forces. In Brown and Ferguson (1980), the distribution of stiffness and yield strength have been investigated. Accordingly, we estimate the force corresponding to each layer of the bone in order to provide users with a tangible force, while penetrating through the different layers. However, the acquired sense of the force is not equal to the actual bone stiffness, and it makes the sense of passing through the layers for users. It is worth to say that the determined stiffness for the bone's layers was limited to the haptic device capability. Therefore, since the discrepancy of estimated force feedback does not influence on the DHG, it can be considered as the scaled stiffness of the real value.

**Figure 5:**
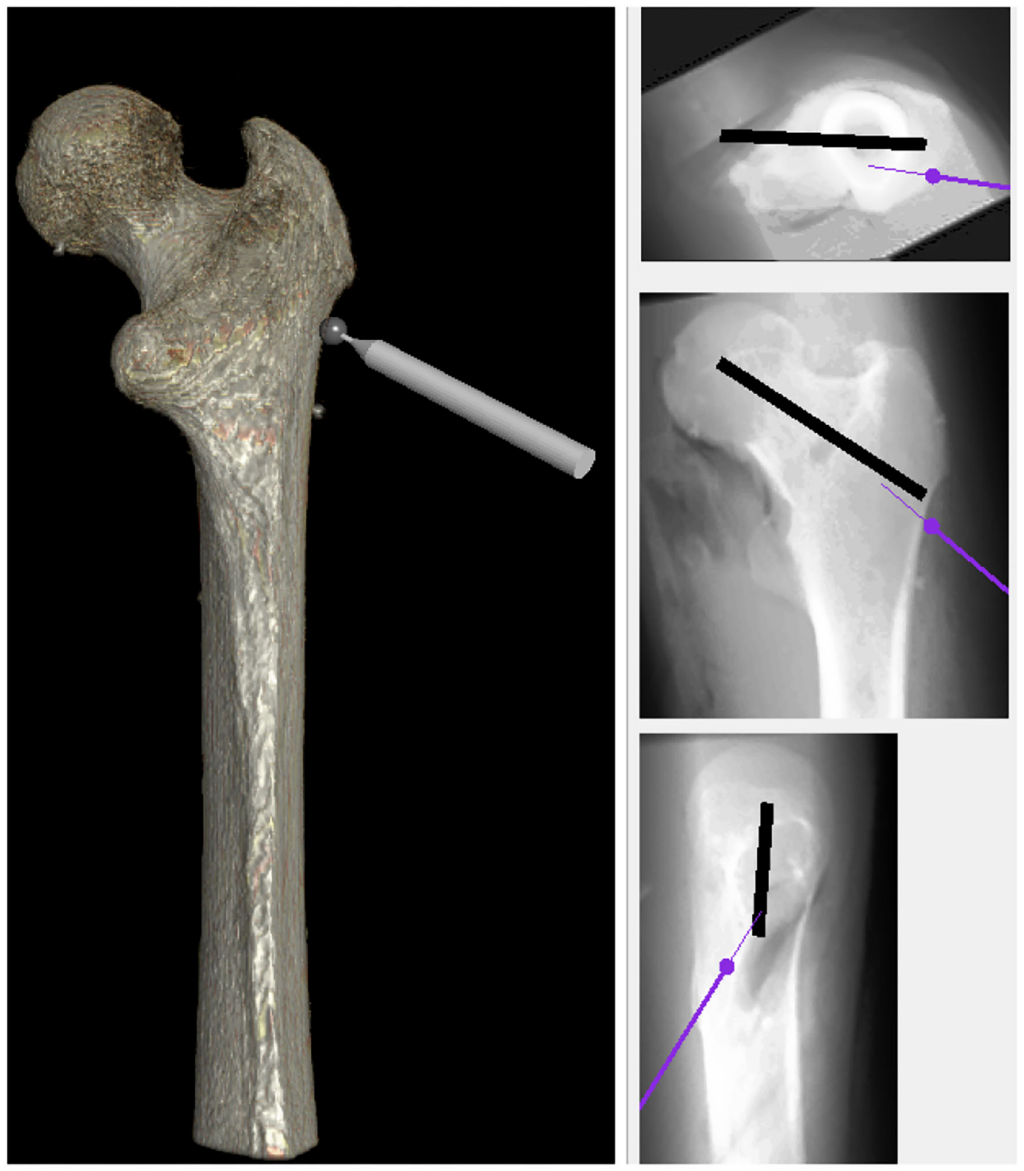
The simulation was equipped with X-ray view for providing users an environment more analogous to the real operating room. Similar to the real operation room, it has three views: the top, front, and side.

Fourth, as shown in Figure 5, it needs to enable the user to take X-ray images from different points of view. In other words, in the real operation room, surgeons capture X-ray images during a surgery to make sure whether the drill traverses through the correct pre-defined path or not. With this in mind, the simulation allows them to have the visualization of X-ray from the bone, analogs to the real operation room, from three views: the top, front, and the side. Eventually, a virtual environment is built to simulate the actual process in the operation room. Apart from provided facilities, the essential data are compiled via the experimental setup. In fact, the simulation strives to imitate real operation rooms as much as possible because it leads expert surgeons to function naturally, thereby capturing more meaningful data. It is good to say that the aforementioned setup in conjunction with the simulation has contributed in other studies in our research Lab at Kettering University (Safavi and Zadeh, 2017; Zahedi et al., 2017, 2019).

### 2.2. Data Preparation

To train the DHG on experts' behaviors, the aforesaid setup was exploited and a drilling path was determined between two points as a specific surgical task. The bone should be pierced from point A to B in the straight direction. Since it was not possible to invite surgeons, six engineering students who were familiar to the area were asked to pretend as our experts' reference and complete the task using the experimental setup. They were required to pass through all the layers of the bone and get to the target point. The participants were guided to perform the task and instructed to complete the task precisely and as quickly as possible. Every subject had 5 min to get acquainted with the experimental setup and then carry out the task. They were allowed to repeat the task as many times as they preferred. Then the best completed task of every attendee were selected based on his/her discretion. In the process of collecting data, the force feedback in x, y, and z axes as well as drill positions, drill penetration depth, the simulated temperature of the drill, and the type of layers in the bone were obtained in the format of a dataset. The data were collected with the frequency of 10 Hz. Eventually, the time-varying data were captured from six completed tasks and *D* ∈ *R*^*n*×9^ constituted the dataset, where *n* is the number of data records. It is noteworthy that for the sake of preserving dynamic properties, the order of records for every task of the dataset was retained.

### 2.3. Creating Models Using Deep Learning Algorithm—DHG

**Figure 6:**
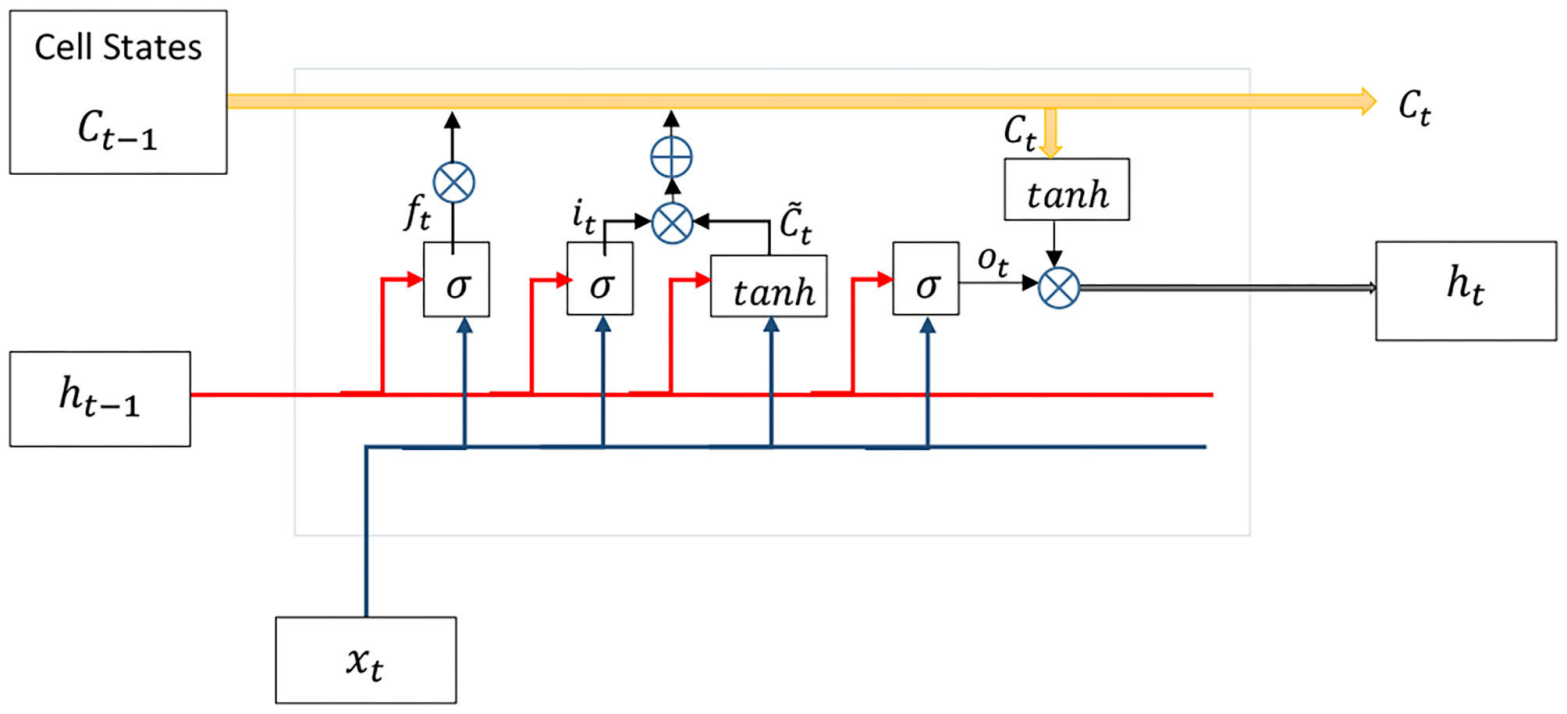
The LSTM unit contains forget, input, and output gates along with cell state (further details in section 2.3).

By taking a closer look at the biological perception of human in performing tasks, it is evident that the action taken in the current time *t* has been concluded by the sequence of actions that happened in the past. This fact can be generalized to the current problem in such a way that “time” plays a significant role in learning expert surgeons' behaviors. On the other hand, learning process evolves during the time by retrying experiences. Accordingly, the DHG learns the experts' demonstrations by consuming the time-varying data, which has been gathered from multiple duplicate tasks (section 2.2). The DHG employs a recurrent neural network instead of a feed-forward one in order to extract the dynamic temporal behaviors of data. In fact, it models gestures of the surgeon in time *t* using a series of data with a specified length. This type of network includes a feedback signal as a loop to consider temporal effects of input data over time. Moreover, the LSTM is a popular architecture for RNNs, which has been proposed to address vanishing gradient problems in Vanilla RNNs (Hochreiter and Schmidhuber, 1997; Hochreiter et al., 2001). Figure 6 depicts the internal structure of LSTM. The aforementioned architecture has been successfully applied in different applications such as the vehicle trajectory prediction for modeling temporal properties of data (Altché and de La Fortelle, 2018).

As mentioned in the previous section, the dataset *D* ∈ *R*^*n*×9^ was prepared while surgeons were carrying out the task. Every feature of the dataset is deemed as an independent data stream, which has been emitted from a separate data source or sensor. From another point of view, as a sensor fusion method, the DHG receives a nine-dimensional vector of data and provides the prediction on force feedback in a three-dimensional vector in its output. Finally, the DHG uses an LSTM as the preferred architecture for the deep recurrent neural network to model the dynamic data.

The DHG is considered to have unequal inputs and outputs size because of the sensor fusion concept. To this end, as described in the following section, a partial modification was applied in the original structure of the LSTM by adding a linear layer (Equation 7). The LSTM's basic is explained in detail, although there are valuable sources in the literature as well.

As a recurrent neural network, the LSTM generates feedback signals via a loop to exert the impact of previous data in the current time instant *t*. Apart from the concept of hierarchical layers in deep neural networks, every unit of the LSTM has four exclusive layers (Figure 6). Cell state *C*_*t*_ keeps past experiences at time *t* and gets updated over time. Forget gate *f*_*t*_ removes data from the memory by using both input *x*_*t*_ and previous output *h*_*t*−1_, while input gate *i*_*t*_ decides which value must be updated:

(1) $$\begin{array}{l} f_{t}=\sigma \left(W_{f}x_{t}+U_{f}h_{t-1}+b_{f}\right) \end{array}$$

(2) $$\begin{array}{l} i_{t}=\sigma \left(W_{i}x_{t}+U_{i}h_{t-1}+b_{i}\right) \end{array}$$

(3) $$\begin{array}{l} \tilde{C}=\tanh\left(W_{\tilde{c}}x_{t}+U_{\tilde{c}}h_{t-1}+b_{\tilde{c}}\right) \end{array}$$

where σ is a sigmoid activation function, *U*_*f*_ and *W*_*f*_ are weight matrices, and *b*_*f*_ denotes the bias vector. A hyperbolic tangent layer calculates new values for update in cooperation with the input gate and then replaces memory *C*_*t*_ with the new values as follows:

(4) $$\begin{array}{l} C_{t}=f_{t}*C_{t-1}+i_{t}*{\tilde{C}}_{t} \end{array}$$

where * denotes the element-wise multiplication. Eventually, the output *h*_*t*_ is calculated by updated memory *C*_*t*_ and the previous output via output gate *o*_*t*_ as follows:

(5) $$\begin{array}{l} o_{t}=\sigma \left(W_{o}x_{t}+U_{o}h_{t-1}+b_{o}\right) \end{array}$$

(6) $$\begin{array}{l} h_{t}=o_{t}*\tanh\left(C_{t}\right) \end{array}$$

It is worth to say that, input $x_{t}\in R^{n}$, weight matrices *W* ∈ *R*^*h*×*h*^ and *U* ∈ *R*^*h*×*h*^, and biases *b* ∈ *R*^*h*^. *n* is the size of the input vector and *h* is the size of the internal memory or cell state, which is defined by designers.

Furthermore, the RNN should be unrolled to establish feedback signals in the internal structure and use the advantages of engaging previous input data in the current time *t*. So, we assume that the LSTM unit output *h*_*t*_ depends on {*x*_*t*−*e*_, *x*_*t*−*e*−1_, …, *x*_*t*−1_} where *e* is the number of the dataset record used for unrolling the DNN. In another way, the loop of RNN is unrolled over *e* latest inputs. Thus, the prediction at time *t* relies on 0.1 × *e* previous seconds of surgeons' behaviors. These unrolled units are defined as the system time steps.

As noted earlier, the DHG fuses multiple features and outputs only force feedback. With this in mind, the size of outputs in the network must be equal to the size of haptic force feedback vector, whereas in the conventional LSTM both input and output vectors are equal in size. To this end, we add a linear output flattening layer with a linear activation function to map inputs into the output space as follows:

(7) $$\begin{array}{l} a=W_{a}h_{t}^{l}+b_{a} \end{array}$$

**Figure 7:**
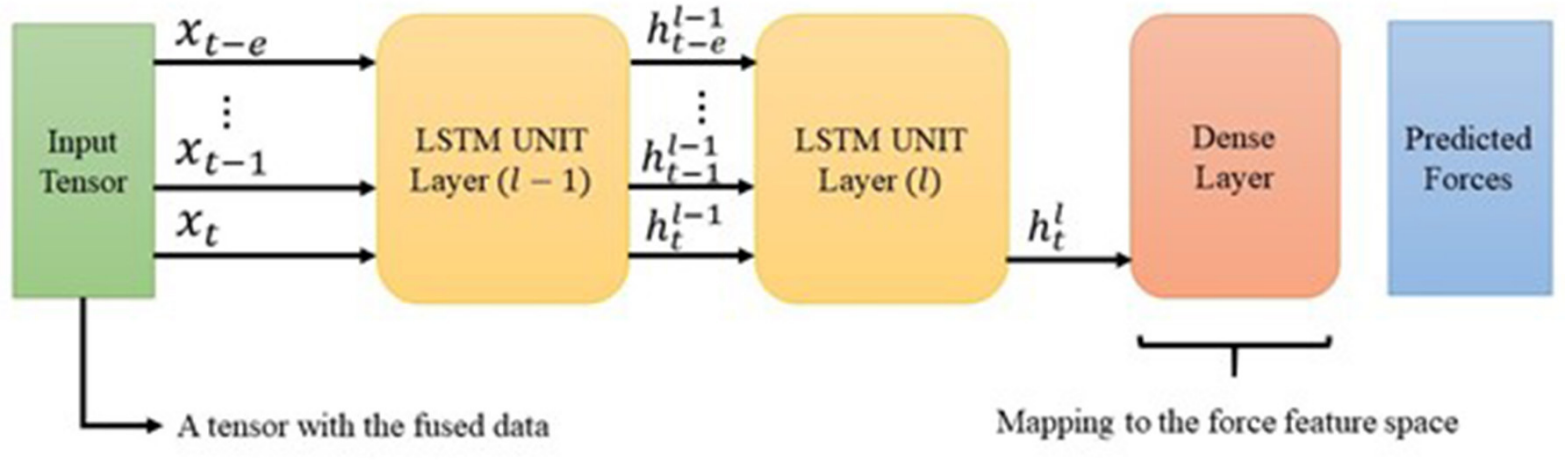
The diagram shows the intended architecture for the DHG. The input is a tensor containing the data from different sources (section 2.2). The LSTM is unrolled over e previously generated data. The cell state h is the input of its corresponding unrolled unit in the next layer. However, using the latest LSTM unit's output, the DHG squeezes the prediction vector through a dense layer. Finally, the output of the network is a vector with three elements corresponding to forces in x, y, and z direction.

By utilizing the above linear transition function, the output at last time step in the last layer is transferred to the force feedback output space. In contrast to classification problems, since the system attempts to generate force values in the output, transition from the feature space *d* ∈ *R*^9^ to force feedback space $r_{t}^{p}\in R^{3}$ is accomplished by a linear regression, where $r_{t}^{p}$ is the predicted force value in time *t* acquired from *e* previous number of the data records. Figure 7 depicts the designed architecture for the DHG. In addition, RMSE is the objective function of the DNN, which attempts to learn the expected output $r_{t}^{ex}$ in a supervised manner as follows:

(8) $$e(r_{t}^{p},r_{t}^{ex})=\sqrt{\frac{\sum_{i=1}^{d}(r_{t,i}^{p}-r_{t,i}^{ex})}{d}}$$

To optimize the above objective function, different optimizer algorithms such as ADAM optimizer can be applied (Kingma and Ba, 2014).

## 3. Evaluation and Discussion

The target of this section is preparing the data, which has been captured in section 2.2 for training models as well as assessing the performance of the DHG in predicting haptic force feedback. In section 3.1, we revise the format of the dataset (section 2.2) and make it compatible with the DHG's input. Finally, we investigate the result in the last subsection. In this work, we did not conduct a human factor study in experiments to examine the performance of the DHG. Instead, we evaluate the DHG based on common assessment methods such as RMSE to show the accuracy of predictions on the reference input. Obviously, haptic force feedback is the reference of the DHG model. It should be noted that we aim to thoroughly analyze the DHG with human-included experiments in a separate study in the future.

### 3.1. Dataset Revision and Model Configuration

**Figure 8:**
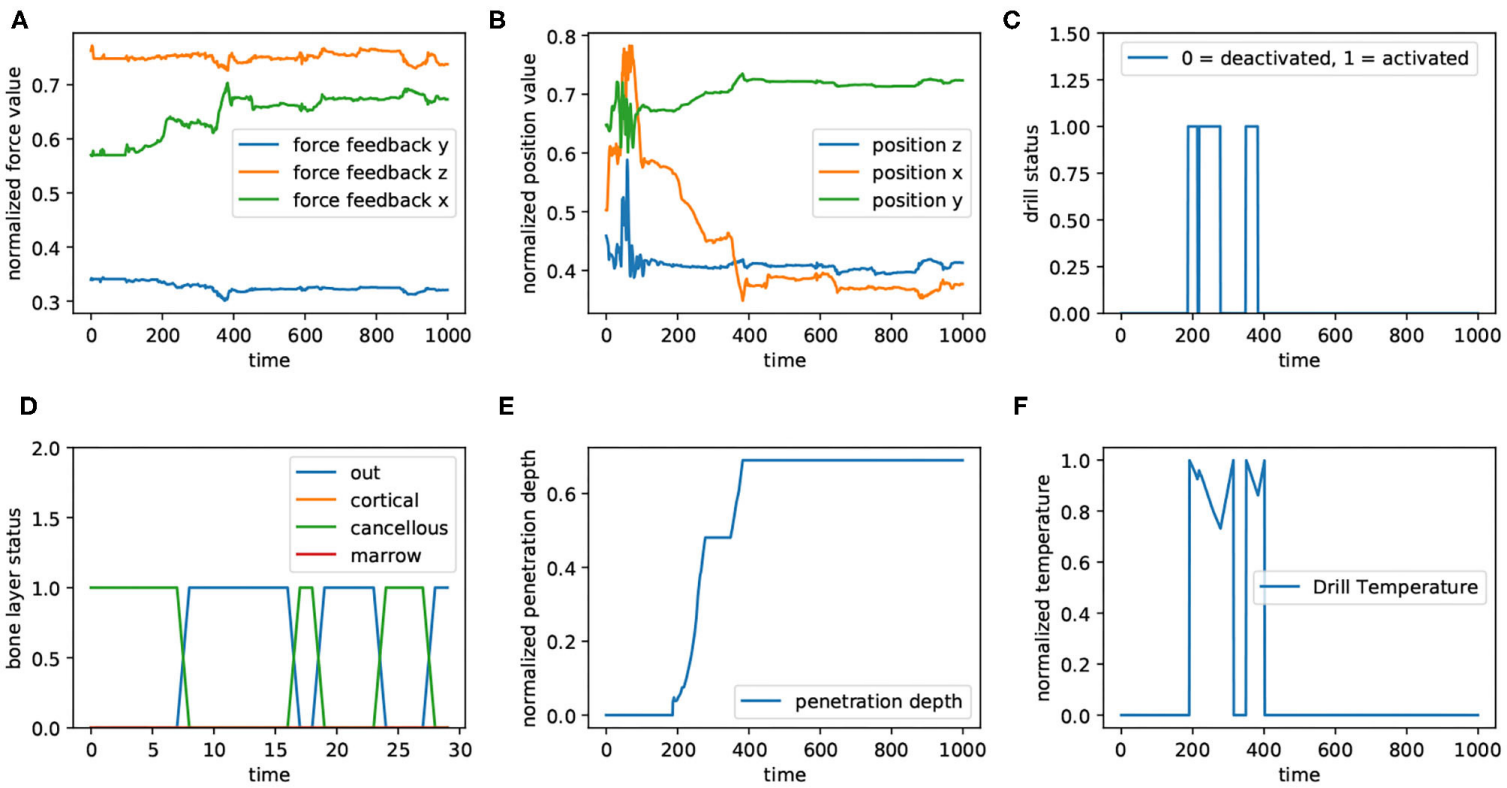
The diagram shows the data captured from different sources in a window of 1,000 data samples. Panels **(A,B)** show the force feedback and positions, receptively. They have been plotted in 3 dimension (x, y, and z). In addition, all values corresponding to force feedback, positions, drill's temperature **(F)**, and penetration depth **(E)** have been normalized (between 0 and 1). Also, for both drilling status **(C)** and bone's layers **(D)**, 0 denotes “inactivated” and 1 means “activated.” Panel **(D)** illustrates the layer of the bone, in which drill is located. For a better visualization in this subplot, the 30 first data corresponding to the aforementioned window are plotted.

As mentioned in the previous sections, we defined a drilling path in the virtual environment and asked six volunteers to drill through that path. As shown in Figure 8, haptic force feedback (Figure 8A), positions (Figure 8B), bone's layers (Figure 8D), depth of penetration (Figure 8E), and drill's temperature (Figure 8F) are the features of the dataset; “Drill's status” (Figure 8C) has not been involved in the dataset, though its correlation with drill temperature is comparable in the figure. This figure represents a time window of captured data from all sensors with 1,000 data records. The values corresponding to force feedback (Figure 8A), positions (Figure 8B), and penetration depth (Figure 8E) has been normalized; in other subplots, 0 means “deactivated” and 1 indicates “activated.” For better visualization, the subplot related to bone's layers (Figure 8D) illustrates the presence of the drill's tip in different layers within a sequence of data with 30 records.

$r_{i}\in R^{9}$ is defined as the *i*th record of the prepared dataset, which is sorted by time. To convert the dataset records to a compatible format for an unrolled network, a modification is needed in such a way that every input in time instant *t* should be a set of *e* previous data records. Accordingly, the new dataset contains the records as follows:

(9) $$\begin{array}{l} nd=\left\{rn_{1}=r_{1:e},rn_{2}=r_{2:e+1},\ldots ,rn_{i}=r_{i:e+i-1}\right\} \end{array}$$

where $rn_{i}\in R^{e\times n}$, *e* is the time step and *t* = *ld* − *e* + 1 (*ld* is the length of dataset before conversion). It is worth to point that every input sample *rn*_*i*_ that encompasses data corresponding to *e* previous data should be mapped to the output space of *e* + 1. In this work, the dataset explained in section 2.2 was converted to the new format with the time step *e* = 20 with the aim of being compatible with the training phase. In fact, the system produced haptic force feedback in the current time using 20 previous data samples. Eventually, the dataset was divided into training and test sets without shuffling (Table 1).

**Table 1:** The result obtained from different configurations of the DNN.

**Configuration**	**Input size**	**Output size**	**Time steps**	**Layers**	**Memory**	**Training sample**	**Test sample**	**Batches**	**RMSE**
1	9	3	20	1	128	9,133	1,442	50	0.0551
2	9	3	20	2	256	9,133	1,442	50	0.0249
3	3	3	20	2	256	9,133	1,442	50	0.0335
4	9	9	20	2	256	9,133	1,442	50	0.0626

*The configuration related to the sensor fusion is shown in the first and second row. Increasing both memory and layer of the network caused to improve the DHG's performance. The superiority of the DHG with a sensor fusion setup (second row) is obvious in comparison with the third and last row. The third setup is the reproduction of the LSTM configuration used (Khatami et al., 2017)*.

### 3.2. Result and Discussion

We complied a dataset containing the demonstrations of six volunteers during accomplishing a drilling task. These data are regarded as the reference behaviors for the input in the training phase. The DHG is supposed to mimic the gestures and act as a professional surgeon in that specific task. In other words, the DHG predicts appropriate force feedback signals in every time instant *t*. The more accurate prediction on the force feedback causes to obtain the more authentic discrepancy between the gestures of novices and experts (Figure 1). For better clarification, there are two types of signal: haptic force feedback and haptic guidance force. The DHG learns how to anticipate the haptic force feedback in different situations. In fact, it assumes that if the DHG is capable of imitating the reference force feedback properly, then it is possible to make a meaningful haptic guidance by extracting the difference between the output of the DHG and the emitted force feedback, while a user (novice) performs a task.

Hence, we set four different configurations of the DHG so as to evaluate the performance of predictions. Table 1 represents the throughput of models. Also, we reproduced the proposed architecture of the LSTM in Khatami et al. (2017) in order to compare the DHG with one of the latest work in the literature. All configurations consumed a same training and test set and the networks were unrolled in 20 time steps with 50 samples of data in each data batch. The size of memory or neurons for the LSTM units is listed for every configuration. Moreover, all configurations were trained in 10,000 epochs and the learning rate was 0.004.

Configuration 1 had 128 memory size through a one-layer LSTM network. The aim of this setup was mapping the input vector of size nine to an output vector with three elements related to the haptic force feedback prediction. Configuration 2 is the intended architecture for the DHG. This setup reached to the best result in comparison with the others. Figure 7 demonstrates the architecture of the DHG. The prepared data (section 3.1) is fed to an LSTM, which is unrolled over *e* = 20. Every hidden state of the unrolled unit enters to another LSTM unit in layer 2. In this unit, only the output of the hidden state in time t goes to a dense layer. Since the DHG aims at estimating the forces as a regression problem, the activation function for the dense layer is a linear one.

As mentioned earlier, to compare the influence of the sensor fusion (DHG) with the conventional multi-dimensional time series prediction in providing haptic force feedback, we set a DNN to anticipate force values in three axes while receiving the same feature vector (force feedback) solitary in the input. Configuration 2 is the implementation of with the same dataset and layers (Khatami et al., 2017). Comparing configurations 2 and 3 of Table 1, it can be seen that the sensor fusion setup has had a positive impact on reducing the error of force-feedback predictions. In fact, configuration 2 (Khatami et al., 2017) has hypothesized that the applied forces in the past is the only parameter, which influences on the currently yielded haptic force feedback. From that perspective, some effective parameters in the surgery such as time of completion, drilling speed, and tissue layer types should be assumed as fixed values or in some way, they have been overlooked.

In contrast, the DHG has attempted to include those parameters in the process of force estimation so as to investigate the effect related to not only physical properties of the bones but also the status of the actuator and its workspace environment on forces. In reality, the expert surgeons' maneuver in time *t* not only depends on the previously taken actions, but also is influenced by the status of the drill and environment it pierces through. The last row (configuration 4) shows the RMSE while the DHG was configured to predict features as same as the input vector. As mentioned before, this is the conventional multi-dimensional time series. Although the output vector contains force feedback, drill's positions, temperature, bone's layer, and penetration depth, the RMSE was computed just by using the elements of output vector related to the force feedback data. Finally, we showed that not only a deep recurrent neural network is capable of learning surgical gestures aptly but also having a sensor fusion mechanism through the network structure can impact on the prediction positively.

**Figure 9:**
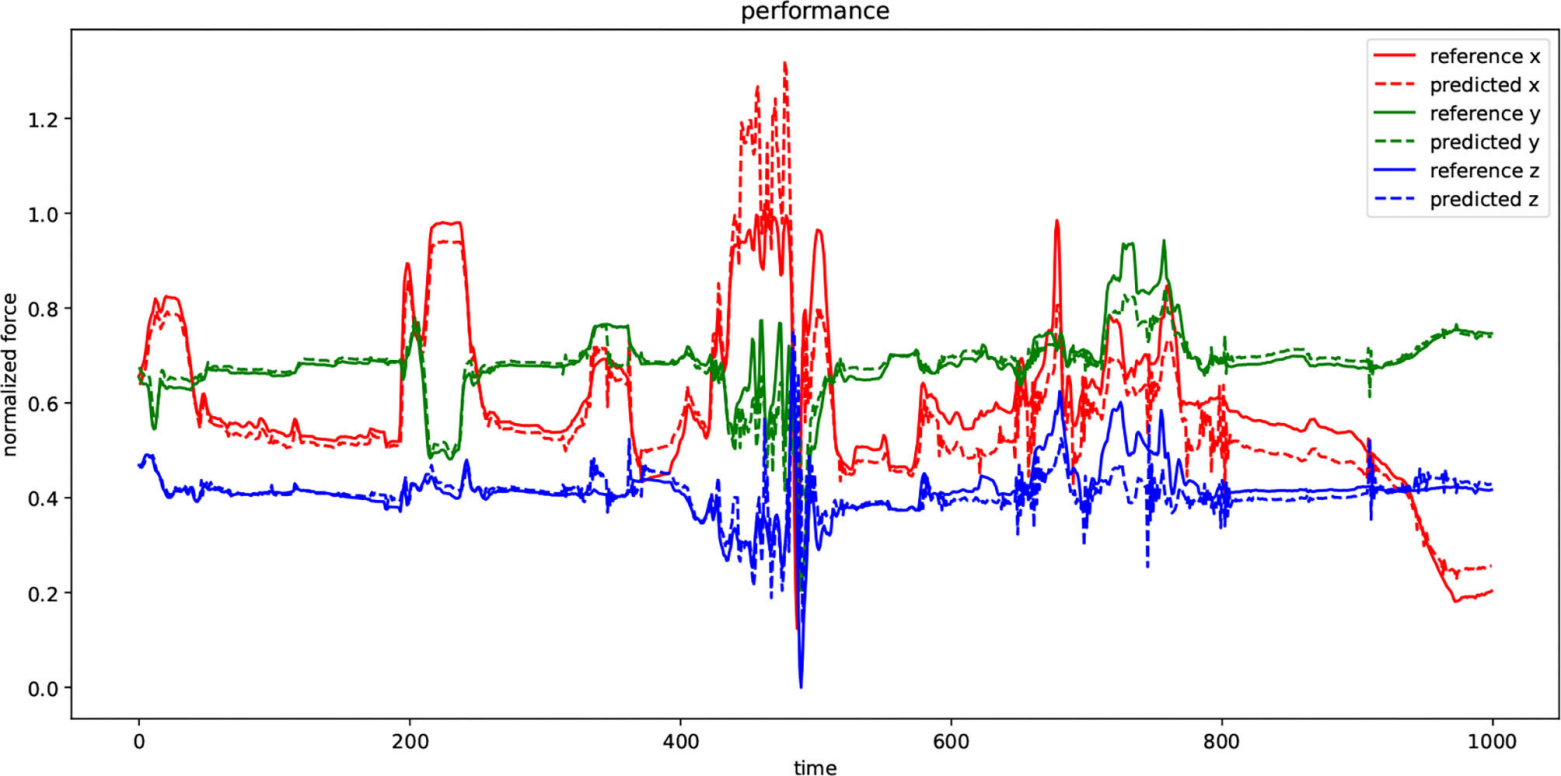
The diagram depicts the performance of the DHG in a specified window with 1,000 data samples. Every dimension of force-feedback signals (x, y, and z) has a reference and a predicted demonstration. The value of the references has been normalized and also the model has been trained by normalized training set as well. The DHG makes predictions on force feedback in every time *t* using 20 previous data samples.

Figure 9 visualizes the performance of the DHG while using second configuration in Table 1 to predict the unforeseen test data. There are two types of signals in the plot: reference and predicted. Every dimension of a force-feedback reference (x, y, z) was normalized. The plot demonstrates the prediction of the DHG for every reference of force-feedback dimensions in a specific time window with 1,000 sample. In other words, it was fed by 20 previous data samples for making anticipations on force feedback in every time *t*. Although the reference signal in both test and training set was normalized, in some points the prediction has exceeded above 1 because of generalization that causes model to have margin around actual data and avoid facing with over-fitting.

It is worth noting that to use predicted guidance signals in the haptic device it should be denormalized regarding to the minimum and maximum of the samples in the dataset prior to dividing data into test and training sets. Estimating force feedback values in the data preparation phase along with normalization and denormalization process may cause to reduce the accuracy of trained models in practice. However, having a more precise guidance signal leads to effectively instruct trainees, but using directions of the force regardless of its value can make an acceptable feeling of haptic feedback, whenever a distraction occurs. All in all, the DHG is a modified version of RNNs with an LSTM architecture, which is a general solution for any problems using time-varying data. Since most of surgical tasks are carried out by surgeons' movements and their corresponding data has similar properties to our dataset, the DHG can be generalized to other specific surgical tasks as well.

## 4. Conclusion

In this work, we presented a deep learning based system in order to explore improvements in the performance of surgeons in surgical drilling operations. The proposed system strives to enhance the skill transfer system for instructing surgeons either the experts or novices via generating haptic guidance signals during the surgery. The system can address the limitations due to especial circumstances such as the COVID-19 pandemic, in which trainees cannot practice surgical tasks. This was achieved by designing a deep recurrent neural network with an LSTM architecture that models the behavior of experts. As a sensor fusion method, the DNN was trained using the data emitted from different sources such as drill's temperature, penetration depth, and the type of bone's layer. This led to have a robust model, which predicts demonstrations precisely. Since the experimental setup is not equipped with a force sensor, the simulation estimates different data values such as stiffness based on the physical properties of simulated bones. Finally, the experimental result showed that the proposed system was able to predict accurately haptic force feedback. Although the proposed method performed effectively in the evaluations, we did not apply the method in a human factor study. In fact, the significant purpose of this work was to inspect the possibility and performance, mostly by regular machine learning methods. Therefore, as a future work, we intend to exert the method in a practical experiment and examine it by surgeons.

## Data Availability Statement

The datasets presented in this article are not readily available because the data belongs to the Reach Lab at Kettering University. Requests to access the datasets should be directed to Mehrdad Zadeh, mzadeh@kettering.edu.

## Ethics Statement

The studies involving human participants were reviewed and approved by The Kettering University Institutional Review Board (IRB protocol 2012.05). The patients/participants provided their written informed consent to participate in this study.

## Author Contributions

PF proposed and implemented the method and also wrote the paper. JD was the supervisor of the research lab as well as this project and defined the problem and contributed in the solution. MZ setup along with the data was provided by him. In parallel, he was the research co-supervisor and also contributed in the solution. All authors contributed to the article and approved the submitted version.

## Conflict of Interest

The authors declare that the research was conducted in the absence of any commercial or financial relationships that could be construed as a potential conflict of interest.
