# Corrigendum: Deep Learning-Based Haptic Guidance for Surgical Skills Transfer

**DOI:** 10.3389/frobt.2021.691570

**Published:** 2021-05-05

**Authors:** Pedram Fekri, Javad Dargahi, Mehrdad Zadeh

**Affiliations:** ^1^ Mechanical, Industrial, and Aerospace Engineering Department, Concordia University, Montreal, QC, Canada; ^2^ Electrical and Computer Engineering Department, Kettering University, Flint, MI, United States

**Keywords:** deep learning, recurrent neural network, LSTM, haptic, force feedback, bone drilling, surgical skill transfer, COVID-19

In the original article, there was a mistake in Figure 7 as published. The figure included “Dropout”, which was not used in our reported results but can be applied to the “Dense Layers” before the output layer. Since we have not utilized “Dropout” in the reported results, the corresponding information has been removed from the caption of Figure 7 and a correction has been made to the **Evaluation and Discussion** section, subsection **Result and Discussion, Paragraph 3**:

“Configuration 1 had 128 memory size through a one-layer LSTM network. The aim of this setup was mapping the input vector of size nine to an output vector with three elements related to the haptic force feedback prediction. Configuration 2 is the intended architecture for the DHG. This setup reached to the best result in comparison with the others. Figure 7 demonstrates the architecture of the DHG. The prepared data (section 3.1) is fed to an LSTM, which is unrolled over *e* = 20. Every hidden state of the unrolled unit enters to another LSTM unit in layer 2. In this unit, only the output of the hidden state in time t goes to a dense layer. Since the DHG aims at estimating the forces as a regression problem, the activation function for the dense layer is a linear one.”

The corrected Figure 7 and caption appear below.

**FIGURE 7:**
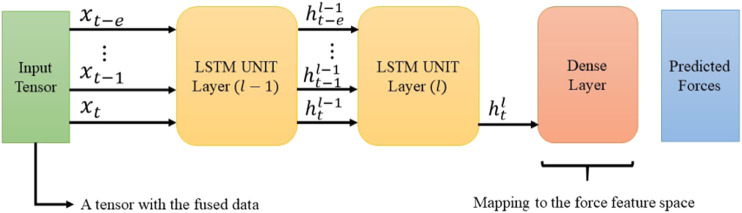
The diagram shows the intended architecture for the DHG. The input is a tensor containing the data from different sources (section 2.2). The LSTM is unrolled over e previously generated data. The cell state h is the input of its corresponding unrolled unit in the next layer. However, using the latest LSTM unit’s output, the DHG squeezes the prediction vector through a dense layer. Finally, the output of the network is a vector with three elements corresponding to forces in *x*, *y*, and *z* direction.

The authors apologize for this error and state that this does not change the scientific conclusions of the article in any way. The original article has been updated.

